# Conversations between self and self as Sigmund Freud—A virtual body ownership paradigm for self counselling

**DOI:** 10.1038/srep13899

**Published:** 2015-09-10

**Authors:** Sofia Adelaide Osimo, Rodrigo Pizarro, Bernhard Spanlang, Mel Slater

**Affiliations:** 1 Event Lab, Faculty of Psychology, University of Barcelona, Barcelona, Spain; 2 ^2^Institució Catalana de Recerca i Estudis Avançats, ICREA, Barcelona, Spain; 3 ^3^Department of Computer Science, UCL, UK

## Abstract

When people see a life-sized virtual body (VB) from first person perspective in virtual reality they are likely to have the perceptual illusion that it is their body. Additionally such virtual embodiment can lead to changes in perception, implicit attitudes and behaviour based on attributes of the VB. To date the changes that have been studied are as a result of being embodied in a body representative of particular social groups (e.g., children and other race). In our experiment participants alternately switched between a VB closely resembling themselves where they described a personal problem, and a VB representing Dr Sigmund Freud, from which they offered themselves counselling. Here we show that when the counsellor resembles Freud participants improve their mood, compared to the counsellor being a self-representation. The improvement was greater when the Freud VB moved synchronously with the participant, compared to asynchronously. Synchronous VB movement was associated with a much stronger illusion of ownership over the Freud body. This suggests that this form of embodied perspective taking can lead to sufficient detachment from habitual ways of thinking about personal problems, so as to improve the outcome, and demonstrates the power of virtual body ownership to effect cognitive changes.

Inner speech (talking to yourself) is considered a critical component of self awareness^1^ and self-related processing^2^. Inner speech may consist of positive or negative self evaluations (if not neutral), which have been shown to activate different brain regions^3^. Moreover sub-vocalisations follow the same pattern as if the person were actually speaking out loud, for example, following regional accents^4^. But when talking to yourself, to whom are you talking? In this paper we introduce a method that externalises a representation of the self to whom you are talking, and additionally shows how you can occupy the body of that self and talk back—to yourself—thus engaging in a genuine self-conversation. In the experiment we describe, the term ‘counsellor’ refers to the externalised self-representation to whom you talk and the issue under discussion is the resolution of a personal problem.

In order to achieve this we exploit the capability of immersive virtual reality (IVR) to embody people in alternate body representations. In particular we address the question as to whether the *form* of the counsellor body has an impact on self-knowledge demonstrated by positively or negatively influencing the mood associated with the participant’s personal problem. Our general idea is that when the participants (all males) have a body ownership illusion over a counsellor body that represents another person this will allow them to find a more satisfactory outcome, resulting in a positive influence on their mood, compared to when the counsellor body is a lookalike representation of themselves.

A body ownership illusion is a perceptual illusion that an external object or surrogate whole body has been incorporated into the body representation of the person concerned. A famous example is the rubber hand illusion (RHI)^5^ where a rubber hand seen on a table in front of the subject is seen to be stroked synchronously with felt touches on the corresponding, out-of-sight, real hand. In about 80% of subjects after as little as 15 s of this stimulation proprioception drifts to the rubber hand which feels as if it is the subject’s real hand^6^. If the rubber hand is attacked then there are corresponding physiological^7^ and brain activation^8^ responses. Asynchronous stimulation does not lead to these results. A similar technique, relying on visuotactile integration between tactile stimulation seen on the surrogate body and corresponding stimulation felt on the real body, has been used for whole body illusions, whether out-of-body^9^,^10^, or with respect to ownership of a plastic manikin body seen from first person perspective (1PP) through video delivered via a head-mounted display (HMD)^11^.

IVR provides a particularly powerful method for the production of such body ownership illusions and the equivalent illusion to the RHI has been demonstrated using this^12^. With respect to the whole body participants wearing a head-tracked stereo HMD can, when they look down towards their real body, see a virtual body that is spatially congruent with their real body, thus visually substituting it, which can also be reflected in a virtual mirror^13^. When the virtual hand of the body is threatened there is corresponding and appropriate cortical activity^14^. Moreover a HMD together with a full body motion capture system can be used to provide visuomotor synchrony, where the virtual body moves in correspondence with real body movements^15^—a powerful method to induce not just body ownership but a strong sense of agency over the virtual body^16^,^17^.

Several recent results have pointed towards the likelihood that when we have an illusion of body ownership over a body different to our own this can change aspects of our perception, attitudes, behaviours and self-identity^18^,^19^. For example, embodiment of adults in the body of a young child results in their overestimating object sizes and a shift of implicit attitudes about the self towards being child-like, compared to embodiment in an adult-shaped body of the same size^16^. Similarly it has been shown that when Caucasian people have an ownership illusion over a dark-skinned body^20^ or body part^21^,^22^,^23^ there is a subsequent reduction in their implicit racial bias, that does not occur as a result of embodiment in a light-skinned body (or body part). Embodiment of light-skinned people in a dark-skinned casually dressed body results in much greater body movement while playing the drums than embodiment in a light-skinned formally dressed body^24^. The illusion of ownership over a body that unexpectedly speaks can lead to illusory self-attribution of the speaking and a change in fundamental frequency of the participant’s subsequent speaking voice towards that of the surrogate body—in other words a false sense of agency^17^.

In the research reported in this paper we embodied participants alternately in two virtual bodies such that they could have an extended conversation with themselves. One body represented themselves and the other a counsellor with whom they would discuss a personal problem. While embodying their own body (lookalike) representation they described the problem. They then transferred to the counsellor body, and from that perspective saw and heard their lookalike body describing the problem, and then gave some insight into how the problem might be solved. They would then transfer back to their own lookalike body and look at and listen to the counsellor body giving them the advice, and then respond to it. If they chose to, they could then once again see and hear this response from the perspective of the counsellor body, and again respond to it. This process of switching between the lookalike and counsellor body continued until the participant decided to stop.

In one condition of the experiment the counsellor body was a representation of Dr Sigmund Freud. In another condition the counsellor body was a duplicate lookalike representation of the participant. When the participant heard himself speak back as Freud the voice pitch was lowered so that it no longer sounded exactly like the participant speaking (see Methods). The setup is illustrated in Fig. 1, and the particular reason for choice of Freud is described in Methods.

In order to achieve this participants wore a wide field-of-view head-tracked HMD, and a motion capture suit (see Methods). When they donned the HMD they were in a room with a mirror towards their left side, and initially saw from 1PP a representation of their real body—both by looking down towards themselves and in the mirror. As they moved their real body the virtual body moved synchronously through the real-time motion capture. On the other side of the room (3 m distant) they saw the counsellor looking towards them—either the Freud body or a duplicate of their own lookalike body (depending on condition). As they talked their voice and body movements were recorded. Hence when their 1PP viewpoint was transferred to the other body, those recordings were played back so that they saw and heard their own previous explanations (see Supplementary Video S1).

Our specific idea was that body ownership over the Freud body would afford participants realising solutions to their problem that they could not reach while embodied in their lookalike body. In other words being in another body would give them access to problem solving resources beyond what would be possible while trapped in the confines of their own body and their normal way of thinking. The different body would give them the opportunity for a new perspective, both literally (they would of course see themselves from a different perspective) and operationally (the body associated with this different perspective representing another person, in this case strongly associated with therapy).

In order to explore this idea we conducted two conceptually distinct experiments. The first (experiment 1) was a within-groups design (n = 12). Each participant experienced two trials separated by one week. Each week they discussed a different problem with a counsellor, but one week the counsellor was the Freud body and the other week it was the lookalike body (Self), counterbalanced in order. In this experiment there was visuomotor synchrony, so that the body moved according to the movements of the participant. Hence we also refer to this as the synchronous (Sync) condition. The purpose of this experiment was to test the hypothesis that body ownership of the Freud body would result in participants improving their mood more compared to being in the Self body.

Another 10 participants were recruited who all experienced the Freud body as counsellor but always with visuomotor asynchrony while in the Freud body. We refer to this as the asynchronous (Async) condition. The visuomotor asynchrony was achieved by using a pre-recorded animation applied to the Freud character. However, when the participant saw the replay of the counsellor giving advice, the true movements made during the counsellor embodiment were replayed. This defines the second conceptually distinct experiment (experiment 2) where the counsellor was always the Freud body, and the comparison was between visuomotor synchrony (n = 12 from the first experiment in the Freud condition) and visuomotor asynchrony (n = 10 in the Async condition). Hence this was a between groups design. The only difference between the two conditions was visuomotor synchrony or asynchrony while embodied as Freud. From previous experiments^16^,^17^ we expected that the asynchronous condition would result in considerably lower subjective rating of the illusion of body ownership than the synchronous condition. Hence this experiment was to test the hypothesis that it was specifically the degree of body ownership of the Freud body that would account for the results—that the mood after the experiment would be more likely to be improved compared to before in the synchronous than in the asynchronous condition.

Body ownership was assessed for the counsellor body through two questions used in previous studies^16^, referred to as *MeDown* and *MeMirro*r in Table 1. The question on agency (*MyMovements*) was to test the functioning of the synchronous and asynchronous conditions. The *LikeMe* question was for two reasons. The first was only to test how well the lookalike virtual character represented the participant from his own point of view. The second was that body ownership illusions have been reported to evoke a further illusion of the participant reporting that the surrogate body resembles themselves in appearance^25^, and therefore we wished to examine whether this might occur for the Freud body in the synchronous condition.

To combine both measures (*MeDown* and *MeMirror*) into one overall score we used the method of polychoric principal components analysis to produce a new combined variable that we refer to as *MyBodyPCA*^26^. (This gives scores almost identical to the mean of the two, and results reported below are not different using the mean).

When participants arrived to the experiment, after reading and completing all ethical approval documents they were asked to think about a personal problem that they would like to resolve. They then assessed this using a Subjective Units of Discomfort scale ranging from 0 (peace, serenity, …) to 10 (insupportable,…). For ethical reasons problems with a ranking higher than 4 were not accepted (something that was bothering them that could not be ignored but which can be managed although giving a bad feeling). We refer to this variable as *SUDS*, which was used as a screening rather than as a response measure.

Mood was assessed using the Profile of Mood States (POMS) instrument^27^ a method widely used in psychophysiology and medical sciences (e.g. dietary weight loss^28^, depression^29^, quality of life with cancer^30^ as recent examples). The POMS questionnaire was administered before the experiment and again afterwards. The measure of interest is the difference between the post experimental total score and the pre-experiment total score (*dPOMSTotal* = *PostPOMS*—*PrePOMS*). The more negative the values the greater the mood improvement.

A second instrument was the Self Assessment Manikin^31^. This is a pictorial technique to directly measure pleasure and arousal on two 9 point scales ranging from 4 (most happy; intense) to -4 (most unhappy; least intense). We refer to the first as *SAMHappy* and the second as *SAMIntensity*. This method is widely used in psychophysiology and neuroscience (e.g. maternal oxytocin responses to infant cues^32^, binge eating disorders^33^, and emotion recognition based on physiology^34^). The SAM instrument was given before the experiment and again afterwards, and our measures of interest are *dSAMHappy* and *dSAMIntensity* which are the post minus the pre experiment measures.

Several other pre- and post-questionnaires were given relating to participants’ overall evaluations of the experience and these are discussed in full in Supplementary Tables 1–4.

Overall 22 male participants were recruited with mean ± SD age 24 ± 5. On a range of demographic variables there were no significant differences between the groups (Supplementary Table 5). The experiment was approved by the *Comisión de Bioética de la Universitat de Barcelona* and carried out in accordance with that approval. Participants gave written informed consent.

## Results

### Body ownership illusion

Figure 2 shows the results by condition for the body ownership and agency related questions (Table 1). It is clear that all the scores are substantially higher in the synchronous than in the asynchronous condition. *MyMovements* verifies the success of the real-time mapping of body movements to the movements of the virtual character—this was perceived by participants as their own body movement (the interquartile range is between 6 and 7 with the median at 7 in the synchronous condition compared with an IQR between 1 and 3 and median of 1 in the asynchronous condition). Overall in the synchronous condition the median scores are high (6 out of the maximum of 7) for *MeDown* and *MeMirror*. The lookalike virtual counsellor was perceived as looking physically like the participant (*LikeMe*) in the synchronous condition but not in the asynchronous, and in the synchronous condition even the Freud counsellor has a median score of 4 (though a large IQR) compared with 1 in the asynchronous condition.

From Fig. 2 it is clear that there are no significant differences in experiment 1 (synchronous condition, counsellor is Self or Freud) for *MeDown*, *MeMirror* and *MyMovements*. For *LikeMe* the Wilcoxon paired signed rank test returns P = 0.008 (two-sided) for the hypothesis that the difference between the two distributions (Self, Freud) has 0 median. Hence we would conclude that the Sync&Self condition results in greater *LikeMe* scores than the Sync&Freud condition. (Note that one questionnaire result was missing so that n = 11 for these comparisons).

For the between groups experiment we compare the Freud counsellor representation between the synchronous and asynchronous conditions. For *MeDown*, *MeMirror* and *MyMovements* the differences are significant (P = 0.003, 0.015, 0.0001 respectively Wilcoxon rank sum tests). For *LikeMe* the difference has P = 0.110 indicating that even with the Freud body in the synchronous condition there may be some propensity to evaluate the virtual body as looking like the participant more than in the asynchronous.

### Comparison of Pre- and Post-Measures of Mood and Happiness

Figure 3 suggests that under all conditions on the average the POMS scores decreased as a result of the experimental intervention and the SAMHappy scores increased, showing an overall improvement of mood and happiness. (There are no differences in SAMIntensity). Comparing *PrePOMS* with *PostPOMS* in the Async condition the difference is significant (Wilcoxon signed-rank test, P = 0.005). For POMS Sync, SAMHappy Async and SAMHappy Sync the corresponding significance levels are 0.0001, 0.027 and 0.0003 respectively. It is evident that the differences between pre and post measures are more pronounced in the Sync condition, and we address this in later sections.

### Comparison of counsellors—Profile of Mood State

First we consider the within group comparison of the Freud versus the Self counsellor (experiment 1). We use mixed effects regression with Counsellor as the fixed effect and individual as the random effect (see Methods), with the results in Table 2. This shows an interaction between Counsellor and SUDS. It is reasonable that the change in mood after the experience compared to before might be correlated with the SUDS, since if the original level of discomfort was already low there is not much room for change. The interaction effect shows that this improvement in mood is related to the type of counsellor, and greater for the Freud counsellor. Moreover the marginal difference between the *dPOMSTotal* for Freud and Self (i.e., eliminating the effect of SUDS) has 95% confidence interval -8.02 to -0.14, also indicating that the Freud condition results in a greater decrease in *dPOMSTotal* (corresponding to an improvement in mood) compared to the Self condition.

### Comparison of counsellors—Self Assessment Manikin

Table 3 shows significant main effects of SUDS and Counsellor for *dSAMHappy*. The results suggest that holding all else constant that the Freud counsellor results in a greater improvement in happiness than the Self counsellor. The 95% marginal confidence interval for *dSAMHappy* Freud minus Self is 0.17 to 1.43.

A similar mixed effects regression for *dSAMIntensity* shows that the interaction term is significant (Table 4, note missing observation). In other words the greater the initial discomfort associated with the problem (SUDS) the less the arousal after the experimental counselling experience than before, especially when the counsellor was Freud. Taking the marginal difference between Freud and Self the 95% confidence interval is 0.02 to 1.64.

### Comparison of visuomotor conditions—Profile of Mood State

Here we consider the between groups conceptual experiment 2 where the counsellor was always Freud and the condition was the visuomotor mapping of real movements to virtual counsellor movements (asynchronous or synchronous). Recall that visuomotor synchrony results in much greater levels of subjective body ownership and agency than asynchronous.

In this case we can use standard regression (in fact ANCOVA) of *dPOMSTotal* on Visuomotor (Async, Sync) and SUDS as the covariate. There is no significant main or interaction effect for Visuomotor. However, we can consider instead the phenomenological subjective product of the different visuomotor conditions—i.e., the questionnaire scores summarised in the variable *MyBodyPCA*. Table 5 shows a negative association between *dPOMSTotal* and the interaction between the ownership illusion and SUDS, where the greater level of body ownership the greater the improvement in mood (i.e., smaller *dPOMSTotal*).

### Comparison of visuomotor conditions—Self Assessment Manikin

The regression of *dSAMHappy* on SUDS and Condition is shown in Table 6 indicating an increase in *dSAMHappy* with the synchronous condition. If instead we use the body ownership variable then this becomes clear as shown in Table 7. Whether we use the actual visuomotor condition or the effect of the condition (Body Ownership) it seems that the level of body ownership in the Freud body had a positive impact on the *SAMHappy* response. However, there are no significant main or interaction effects for *dSAMIntensity* on SUDS, Visuomotor, or MyBodyPCA.

### Example conversations

The types of conversations that occurred are illustrated with the following two. In the first example the participant explained to the Freud counsellor that he was having trouble with his boss, that “…his style of communication towards me, that I consider it’s a bit violent, a bit bold-faced, sometimes with lack of manners even, and I find that a bit… it makes me a bit uncomfortable and I find that discouraging for the work I’m doing.” Then embodied in the Freud body the participant replied: “Maybe a possible solution you could try is to talk directly to that person about the subject, because maybe he’s not aware of the way he addresses you and maybe with the fact of you explaining and showing it and how it makes you feel uncomfortable, it could be useful for that person to realize it and maybe solve it.” The participant then embodied back as himself said that he had tried that, and then re-embodied as Freud said “If that is true, then maybe the possibility that you have done something to that person, to your boss, that may have bothered him, that may have been uncomfortable for him can be considered, and he’s responding in that way. But I think the best solution is to sit down and talk it through in a civilized way.” Finally the participant agreed, and ended the session. What is interesting here is the possibility that the participant already knew that he might have done something to upset the boss to call forth the uncomfortable situation, but that this was expressed when embodied as the counsellor.

In the second example a participant explained how much he missed a girl who he had been close friends with but she had moved to study elsewhere. As Freud he replied: “Well, I recommend you to move on because it doesn’t really solve anything. It’s not a huge problem, but it doesn’t help to live in the past. I think you should keep it as a good experience and memory, but not something that keeps you from going on.” The participant, embodied back in his own body replied: “It’s true, it’s very true I shouldn’t be living in the past. You can miss someone a lot, but it doesn’t have to be something that keeps you from going on, from living your life and living the moment, you know, everyone follows his own path and you have to meet new people that gives you something else. You have to live every day at the maximum, enjoying life.” This was the end of the session.

## Discussion

Overall under all conditions the self-counselling experience was positively evaluated by participants (Supplementary Tables 1–4) and tended to improve mood and happiness (Fig. 3). However, experiment 1 showed that the Freud body tended to further enhance these in comparison to the Self body. Experiment 2 showed that the Freud body tended to result in greater mood improvement and happiness in the visuomotor synchronous condition than in the asynchronous condition. Visuomotor synchrony strongly influences the illusions of body ownership and agency. Therefore body ownership and agency specifically over the Freud body tended to have a greater positive influence on mood and happiness than the Self body as counsellor. In what follows we discuss a number of possible explanations for this finding: generalisation of positive self-attributes to an outgroup, perspective taking, self-compassion, similarity with the empty chair technique of gestalt therapy, and the relationship between neural activation due to perspective taking and self processing. Then after discussing how the study needs to be expanded we put it into the context of its potential use in the growing crisis caused by the shortage of provision for mental health problems.

Previous studies on the perceptual, behavioural and cognitive correlates of body transformation have concentrated on embodiment of adult participants in bodies that are representative of a group: for example, children^16^ or a different race^20^,^21^,^23^,^24^. An explanation offered for the reduction of implicit racial bias after embodiment of light-skinned participants in a dark-skinned body is based on the idea of the embodiment generating a perceptual similarity between the self and the outgroup^18^. Briefly, the illusion of body ownership over an outgroup body leads to a physical association (an illusion of resemblance) between the self and that outgroup which in turn leads to the generalisation of positive self attributes towards the outgroup. However, this explanation does not apply to the quite different setup of this experiment since there is no aspect that concerns bias towards an outgroup.

The general improvement of mood could, however, be due to perspective taking—in this case seeing the self from the outside, from another perspective^35^. Perspective taking involves putting oneself in the shoes of another. It is an imaginal and cognitive change of location to the position of another. In our paradigm the participant actually interacts with the self from the perspective of a different location outside of the body, but still embodied in a body. However, since the result is different depending on whether it is the Freud or Self body, the *form* of the body itself must play a role, and so the result cannot simply be only to experiencing the scenario from a different perspective.

This situation of seeing yourself from the outside, irrespective of the form of embodiment, and even discussing with yourself may, however, promote self-compassion where “… there is ‘mental space’ in which to extend oneself kindness and recognize the broader human context of one’s experience”(p88)^36^ This is in contrast with overidentification where “… individuals become so immersed in their current emotional reactions that other aspects of the person—those capable of alternative emotional responses or mental interpretations, for example—are inaccessible…Because one’s awareness is totally consumed by subjective reactions, one cannot step back from the situation and adopt a more objective perspective” (p88). The emotional distance from the problems of the self may reflect the virtual physical distance from the self during the counselling periods. This may explain the overall improvement in mood and happiness due to the experience but it does not explain the differential effects of the Freud and Self embodiments, just as in the same way that embodiment in a child or scaled down adult body both result in overestimation of object sizes, but the child form of body results in almost double overestimation compared with the adult body^16^. In other words if our result were only due to the effects of conversing with oneself from a different locational perspective, then we should have found the same results for the Freud and Self body, but the results of experiment 1 contradicts this.

A practical application of perspective taking is the ‘empty chair technique’ of Gestalt Therapy^37^,^38^. In this method clients in a therapy session typically argue with an imaginary significant other sitting across from them in an empty chair. This may also involve clients eventually sitting in that chair and then experiencing and arguing about the problem from the perspective of the significant other. Typically this method is used to resolve outstanding issues with another person (a parent, a boss, etc). Our paradigm resembles this, and a variant of it could be used for that purpose, but it is not the same, since in our case the other is a counsellor rather than someone with whom there is a problem. The counsellor (whether Self or Freud) is intended to advise on how to resolve the problem rather than being the cause of the problem. However, there may be similar perspective taking mechanisms involved in the operation of both methods which explain why the method works at the general level, but cannot explain the different effects of the Freud and Self body representation.

A previous experiment adds weight to the idea that simply considering the personal problem when seeing one’s own body from a different location (literal perspective taking) is not enough to explain the result. A much simpler version of our paradigm was used to increase self-compassion amongst a non-clinical group of excessively self critical individuals^39^. The method was employed to enable them to indirectly give compassion to themselves^40^. In the virtual reality while embodied in a (non-lookalike) adult virtual body they saw a virtual crying child and delivered a previously learned compassionate speech to the child, that then gradually stopped crying. Afterwards participants were either embodied in the child or in a third person non-embodied condition. In both cases they saw and heard the virtual body that they had previously embodied deliver the compassion speech. The results suggested that those who were embodied in the child body reported a greater degree of self compassion after the experiment compared to before with no significant change in the non-embodied group. In both conditions (embodied in the child or not embodied) participants witnessed their previous embodiment delivering the speech, but the effect of the embodied condition was greater in improving self-compassion than the non-embodied. Hence it must be the embodiment per se that is effective rather than just a change of perspective.

Several studies have shown that perspective taking and self-referential processing are very closely related at the neural level. The key feature that is common to both types of mental activity is the activation of the medial prefrontal cortex (MPFC)^41^. Activations of various subareas of the MPFC have been shown to be implicated in a large number of different aspects of emotional processing about the self and others^42^,^43^. Moreover reflecting upon the self from an egocentric first person perspective results in greater activation of the MPFC than reflecting upon the self from an imagined third person perspective (3PP)^44^. In these types of studies, subjects are typically asked to carry out a task from their normal 1PP or from an imagined 3PP at a different position, or in an avatar body at that position^45^,^46^. Differential neural processing is found from these two different perspectives, though activation of the MPFC seems to be common to both^43^. Furthermore, self-reassurance is positively associated with activity in the VMPFC (ventromedial PFC) compared to engaging in self-criticism.

Our speculation is that when in the counsellor body perspective taking is activated, and the activation of the MPFC may also include the activation of the VMPFC associated with greater self reassurance. Moreover, the self concept is activated. But in the Freud body the ‘self’ is now associated with the attributes of Freud, not the usual self, in common with the explanation of reduced bias when embodied in an outgroup body^18^. All of this together gives the participant access to mental resources that are normally not accessible due to their habitual modes of thinking about themselves. The original finding here is that this generalises to being able to enhance the capability for self-counselling, suggesting the deep and surprising power of body ownership based on bottom up multisensory processing to generalise to higher level capabilities. The mechanism involved remains to be explored with subsequent brain imaging studies.

This experiment cannot address the issue as to whether the result is due to the use of the Freud body specifically, with its strong association with therapy, or whether any other body would have served just as well. We believe, though, that the type of body that would be suitable for counselling is likely to be constrained. For example, it is unlikely that a body associated with negative attributes would be effective. Or, perhaps the content of the counselling would be quite different depending on the body representation used (e.g. embodiment in a body representing high moral standards might lead to a quite different outcome compared to being embodied as an obvious criminal). This speculation is supported by the fact that the choice of Freud as counsellor was, after all, determined by a sample from the population from which the participants were drawn (see Methods). These are fascinating questions that remain open to significant further study.

It should be recalled that in this experiment the participants presented fairly mild personal problems, and that the outcome assessments were only through questionnaires. A further study is needed to consider whether more severe issues might be successfully addressed using this method, going beyond questionnaires to look at actual outcomes, and with long-term follow-up.

Mental health problems account for a large proportion of the world wide disease profile^47^. One study states that one third of all ill health is accounted for by mental illness^48^. In the UK it is estimated that about one in four will have mental health problems each year^49^, with the annual cost of mental health to the UK economy as much as 100 billion pounds.^50^ Yet the provision of mental health help of even the basic psychological ‘talking therapies’ falls far short of requirements. According to a 2014 survey^51^ by the UK Charity Mind of 2000 people who tried to access talking therapies more than 50% were offered no therapy through the National Health Service, and while waiting, almost 70% became more mentally unwell, with 1 in 6 attempting suicide. The UK government has started a programme to try to greatly increase access to such psychological therapies by the year 2020.

Apparently non-severe conditions such as self-criticism or shame can, if not treated, eventually become far more serious illnesses including depression^52^. The method we have introduced shows some promise in being able to help people tackle relatively minor problems through a form of ‘talking to themselves’. The use of virtual reality in psychological therapy has long been well-established^53^, and also the use of intelligent virtual agents as therapists has been employed^54^. However, such approaches are in sharp contrast to the method presented here, which of course does not require any artificial intelligence to drive the therapist, since the ‘therapist’ is actually also the client, embodied in the counsellor body.

Of course transferring to another body, no matter how eminent a person that body represents, cannot by itself give people access to new knowledge that they did not have before. For example, putting someone in the body of Albert Einstein might make them more open to learning physics but it is not in itself going to suddenly make them experts on the general theory of relativity. In the case of personal problems addressed using the method of this paper, however, the situation is different. The participants are already experts on the subject matter of their problem—since it pertains to themselves and their own lives. What they lack perhaps is the ability to step back and be able view the problem from the point of view of being someone else who has some expertise at dealing with problems. This is dissociation of the problem from the self and importantly an association between the (alternate) self and a personal problem-solving ability. Moreover the participants also normally have full access to all the necessary information about their problem, residing in their autobiographical memory. Hence as a simple method to begin to tackle uncomfortable issues in the lives of people our method might prove to be a useful and inexpensive approach prior to the step of accessing psychological therapy. This possibility is feasible today with the advent of high quality immersive virtual reality displays and tracking at consumer prices, and the massive growth expected in this industry in the coming years. Hence conversations between yourself and yourself as Freud (or anyone else) could be a useful and natural first approach in personal counselling.

## Methods

### Procedures

**Figure 1:**
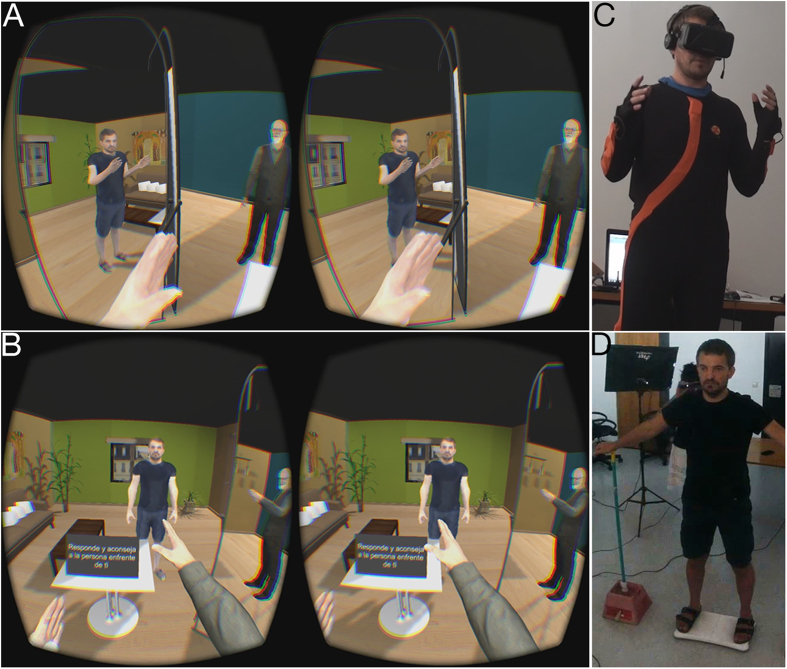
The counselling scenario. (**A**) Stereo 1PP view from the participant in his lookalike body, looking towards the Freud counsellor. He sees himself from 1PP and also in the mirror to his left. (**B**) Stereo 1PP view from the Freud body looking towards the lookalike representation of the participant. He sees himself as Freud from 1PP and also in the mirror to his right. Readers may fuse the two images in each of (**A**,**B**) into one stereo image by crossing their eyes. (**C**) The participant with the HMD and the motion capture suit. Note how the posture is reflected in (**A**) through the motion capture. (**D**) The participant being scanned to make his lookalike body.

**Figure 2:**
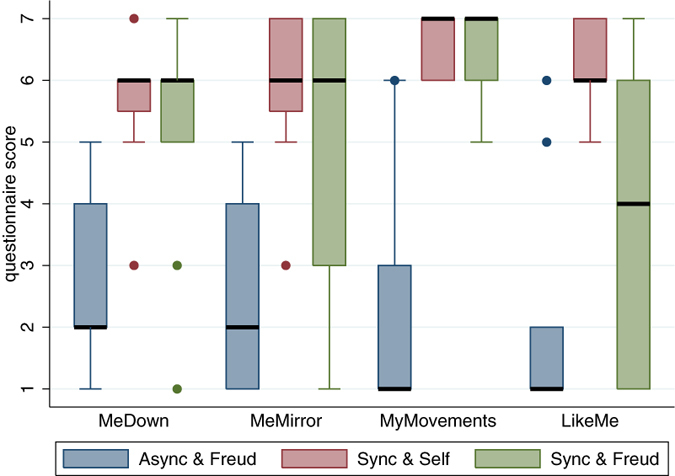
Box plots for the body ownership related questions by condition (Table 1). The medians are shown as the thick horizontal lines, and the boxes are the interquartile ranges (IQR). The whiskers extend from L = max(p_25_−1.5 × IQR, x_1_) to U = min(p_75_ + 1.5 × IQR, x_n_), where p_25_, p_75_ are the 25^th^ and 75^th^ percentiles respectively, and x_1_ and x_n_ are the smallest and largest data points respectively. Points outside this range are shown individually. Async and Sync refer to the asynchronous and synchronous virtual body movements, and Self and Freud refer to the counsellor virtual bodies. n = 10 for Async & Freud, n = 12 for each of the other two conditions.

**Figure 3:**
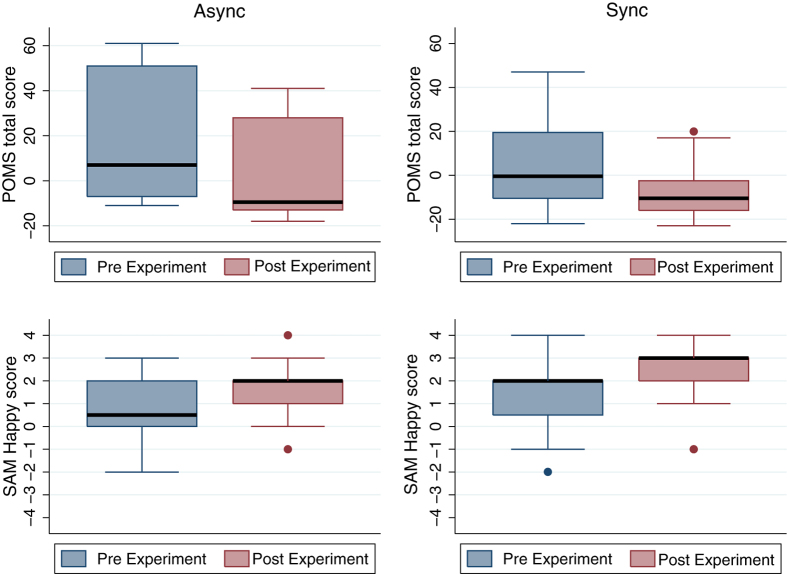
Box plots of pre- and post-experiment measures on POMS and SAM.

Twenty two males were recruited by advertisement around the campus of the University of Barcelona. For both experiments, 1 and 2, participants attended the laboratory and the purpose of the experiment and the procedures were explained to them. If they agreed to take part they completed the ethical consent forms giving written informed consent. They also completed demographic forms recording age, amount computer game playing, and other factors (reported in Supplementary information). Participants reporting having a traumatic past were excluded. Participants were then scanned so that their virtual body likeness could be constructed (see Body Scanning below, and Fig. 1D).

About 40 minutes later they returned to the laboratory (the time in between was to prepare the scanned body for display). They then completed the POMS and SAM questionnaires. They were then asked to think of a personal problem that they would talk about with Freud if given the chance to, and to rate it on the SUDS scale. Participants were explicitly encouraged to choose a problem that was not too severe.

In experiment 1 (within groups comparing between Freud and Self embodiment, n = 12) they would eventually experience two counsellors separated by a week. We refer to the counsellor (Freud or Self) that they experienced the first week as counsellor1 and the one experienced the second week (Self or Freud) as counsellor2. After putting on the virtual reality equipment (HMD and motion capture suit) they experienced 5 minutes of embodiment in counsellor1. This was always with visuomotor asynchrony (the virtual body moved according to a pre-recorded animation). They saw their virtual body both from an egocentric 1PP (i.e., when they looked towards their real body they would see the substituted virtual body) and also in a mirror. They then rated this experience with the questionnaire described in Table 1.

After this they experienced again embodiment in counsellor1 but this time with visuomotor synchrony, and after 5 minutes they gave the ratings of the questions in Table 1. (See the next section for an explanation of the reason for this procedure).

The main part of the experiment then started and they had the interaction with the counsellor as described earlier. First in their own lookalike virtual body they explained the problem, and then they switched to the body of counsellor1 and saw and heard themselves explaining the problem. They then responded to the problem as counsellor1 and on switching back to their original body saw and heard counsellor1 giving his advice, and so on switching back and forth until they decided to finish. The instructions about what they should do after each body change were displayed through a screen on the desk in the virtual environment (Fig. 1B). At the end of the dialogue, while participants were still wearing the HMD and immersed in the virtual environment, they were asked to rate the usefulness of the experience (Supplementary Table 1).

At the end of this session they removed the HMD, answered the POMS and SAM questionnaires, and other questionnaires (Supplementary Tables 2–3). A semi-structured oral interview followed, where the experimenter asked the participant to describe the general impression they had of the dialogue, how the experience of changing perspective and counselling themselves had been, whether the participant thought there were differences between thinking over a problem and discussing it in such a setting, and what kind of problem they thought could be discussed in this setting. Analysis of these recordings is left to a subsequent paper entirely based on techniques for the analysis of qualitative data.

One week later they returned and now expressed their second problem giving it a SUDS rating, and answered the POMS and SAM questionnaires as in the first week. Procedures were then as in the first week, except that counsellor2 was used rather than counsellor1. After taking off the HMD they answered the POMS and SAM questionnaire again. They were then interviewed, during which they were asked to compare the experiences of the two dialogues, choose the counsellor they had preferred (Supplementary Table 4), and describe the difference between the two experiences. They were then debriefed.

For experiment 2 (between groups comparing synchronous and asynchronous) a further 10 participants were recruited. The procedures differed from those described above in three respects. First, the counsellor was always Freud. Second, they experienced the two embodiment phases in the opposite order—first synchronous visuomotor and then asynchronous visuomotor. While in the body of the counsellor during the counselling session they always had asynchronous visuomotor. Third, they only visited once. The purpose was that the results of these 10 were for comparison with the visuomotor synchronous Freud results from experiment 1.

### Assessing Subjective Body Ownership

In both within (experiment 1) and between (experiment 2) groups experiments there is a problem in assessing subjective body ownership^55^. If participants experience an asynchronous condition first, since they have no criterion against which to compare their experience, they may tend to give quite high scores for body ownership based on the fact that when they look down towards themselves they will see the trunk of a virtual body coincident with their own, even if the limbs are not moving the same as their own limb movements. Such first person perspective has been found to be an important factor in the illusion of body ownership^13^,^56^,^57^,^58^. To overcome this problem we used a novel technique where in a training period prior to the start of the main experiment participants had two periods of embodiment in the specific counsellor virtual body that they were going to experience later. If in the main experiment they were going to experience an asynchronous condition, then in their first training trial they would experience a synchronous condition, give their questionnaire scores, and then the asynchronous condition, and again give their questionnaire scores. If they were going to experience the synchronous condition, then trial 1 would be an asynchronous condition and trial 2 the synchronous condition. Their questionnaire responses on trial 2 would therefore always be in the light of their experience in trial 1. The trial 2 scores are used in the analysis of body ownership. (See Supplementary Figs. 1–3 for full analyses of all these data).

### Choice of Counsellor

We could have had the counsellor as an anonymous person. However, then we would have had no control over the level of trust that participants would give to this person based on appearance (for example, accidentally looking like someone with whom a participant had had a bad experience). Therefore we decided that it had to be a figure that everyone would know. Freud was chosen as the counsellor after a number of informal surveys amongst the population from whom the participants were ultimately drawn. 20 people around the campus were asked to choose a famous person that they wished they could talk to about a personal problem. Among the choices the most popular ones were Sigmund Freud, Angelina Jolie and Nelson Mandela. We then asked another 20 males around the campus what type of problem would they talk about with each of these three, and Sigmund Freud turned out to be the popular choice for discussion about a personal problem.

There was also priming for Freud during the experiment, in case participants might not have made the connection between the counsellor and Freud. This was accomplished with a large picture of Freud on the wall of the experimental room. Moreover, when they were asked to choose a problem, they were asked to choose “…a personal problem that they would talk about with Freud.”

### Voice Transformation

When Freud as counsellor spoke the participant’s recorded voice was modified to have a lower pitch. The voice transformation was performed using the SoundTouch Audio Processing Library (www.surina.net/soundtouch), and the operation applied was a pitch lowering by 2 semitones. This had the effect of producing a deeper voice without distorting either the length or the speed of the audio. The pitch lowering value was chosen after an informal survey in which an independent sample (n = 7) recorded their voice and were asked if they recognized their voice after different distortions, ranging from half a semitone to 5 semitones, and whether the distorted voice sounded natural. A distortion of 2 semitones was found to be sufficient to make the voice not recognizable as the participants’ own, but not unrealistically low.

### Materials

The head-mounted display used was the Oculus DK2. This has a resolution of 960 × 1080 per eye, a Field of view 100° nominal, 84° horizontal. It includes a built-in head tracking device that performs sensor fusion at 1000 Hz of gyroscope, accelerometer and magnetometer data (https://www.oculus.com/dk2/).

Participants wore an XSens MVN motion capture suite consisting of the MVN Link 17 tracker tracking suit and MVN Studio software to stream motion data. (https://www.xsens.com/products/xsens-mvn/). The motion replay was performed in a manner similar to that described in Ref. 59.

The computer program was executed on an Asus N750JK laptop with an Intel Core i7-4710HQ CPU, with 8GB of RAM and an Nvidia GeForce GTX 850 M, running Windows 8.1 and DirectX 11. The environment was built with Unity3D 4.5.1 engine and Oculus 0.4.3 SDK.

### Body scanning

Participants were first asked to stand in the middle of an evenly lit room with their arms in a T pose. The experimenter acquired their 3D scan by slowly pointing an Asus Xtion Pro Live RGB-D sensor up and down and around the participant. We used the software Skanect (http://skanect.occipital.com/) version 1.6 to acquire the whole body scan. Participants then sat while the experimenter took close-up scans of their head, of their open mouth and of their arm with a Primesense Carmine 1.09 close range RGBD sensor, which results in improved details of the face and hand geometry and texture. These separate scans were then combined into a single whole body mesh of 10000 triangular faces. The vertices of the body mesh were associated with the bones of a standard skeleton by the Mixamo autorigging service (http://www.mixamo.com/). Head, arms, hands, legs and feet of the virtual body were moved independently by the real time whole body motion capture stream. The mouth of the body could be opened and closed (based on the 3D scans of the participant’s open and closed mouth) and was moved in real time based on the participant’s voice input captured through a headset microphone, approximating the participant’s mouth movements when speaking.

### Statistical methods

We have used the simplest statistical techniques appropriate to the situation. Hence, wherever possible non-parametric tests have been used. This was not possible with the more complex aspects of the design. Hence, results from experiment 1 (within groups) were analysed using a mixed effects model with fixed effect Counsellor and random effects over the individuals. This was carried out using the Stata 13 function ‘mixed’. Experiment 2 was a between groups experiment and therefore analysed with the general linear model (analysis of covariance). In all cases the robust standard errors provided by Stata 13 were used. These are more conservative than normal standard errors (i.e., they are larger) and allow for departures from the assumptions of the statistical model. The two questionnaire variables (*MeDown* and *MeMirror*) were combined using Polychoric PCA^26^ which is a method that specifically accounts for the fact that the variables being combined are ordinal.

## Additional Information

**How to cite this article**: Osimo, S. *et al.* Conversations between self and self as Sigmund Freud – A virtual body ownership paradigm for self counselling. *Sci. Rep.* **5**, 13899; doi: 10.1038/srep13899 (2015).

## Supplementary Material

Supplementary Information

Supplementary Video S1

## Figures and Tables

**Table 1:** Body Ownership and Agency Questionnaire.

Variable Name	Statement
*MeDown*	Even though the body I see might not physically look like me, I feel that the virtual body I see when I look down towards myself is my body.
*MeMirror*	Even though the body I see might not physically look like me, I feel that the virtual body I see reflected in the mirror is my body.
*MyMovements*	I feel that the movements of the virtual body are caused by my own movements.
*LikeMe*	The body I see in the virtual world physically looks like me.

Each statement was scored on a 1–7 Likert scale where 1 indicates no agreement and 7 complete agreement.

**Table 2:** Mixed Effects regression of *dPOMSTotal* on Counsellor and SUDS (Experiment 1).

Term	Coef.	Robust S.E.	P	95% C.I.	Cohen’s *f*^ 2^
Intercept	−19.47	8.36	0.020	−35.87 to −3.08	
Counsellor (Self = 0, Freud = 1)	25.00	12.00	0.037	1.47 to 48.52	
SUDS	2.97	2.24	0.184	−1.41 to 7.35	
Counsellor × SUDS	−9.43	3.97	0.017	−17.21 to −1.65	0.35

Overall goodness of fit Wald χ^2^(3) = 8.45, P = 0.0375. (n = 12 groups with 2 sets of observation per group).

**Table 3:** Mixed Effects regression of *dSAMHappy* on Counsellor and SUDS (Experiment 1).

Term	Coef.	Robust S.E.	P	95% C.I.	Cohen’s *f*^ 2^
Intercept	−0.75	0.68	0.273	−2.08 to 0.59	
Counsellor (Self = 0, Freud = 1)	0.81	0.27	0.003	0.27 to 1.34	0.33
SUDS	0.46	0.22	0.033	0.04 to 0.89	0.10

Overall goodness of fit Wald χ^2^ (2) = 18.17, P = 0.0001. (n = 12 groups with 2 sets of observation per group).

**Table 4:** Mixed Effects regression of *dSAMIntensity* on Counsellor and SUDS (Experiment 1).

Term	Coef.	Robust S.E.	P	95% C.I.	Cohen’s *f *^ 2^
Intercept	−2.24	2.07	0.278	−6.30 to 1.81	
Counsellor (Self = 0, Freud = 1)	7.58	2.88	0.009	1.92 to 13.23	
SUDS	0.43	0.68	0.532	−0.91 to 1.77	
Counsellor × SUDS	−2.25	0.93	0.016	−4.08 to −0.42	0.52

Overall goodness of fit Wald Chi-Squared(3) = 9.82, P = 0.020. (n = 10 groups with 2 sets of observation per group, 1 group with one missing observation).

**Table 5:** Regression of *dPOMSTotal* on SUDS and MyBodyPCA (Experiment 2).

Term	Coef.	Robust S.E.	P	95% C.I.	Cohen’s *f*^ 2^
Intercept	11.49	9.80	0.257	−9.17 to 32.16	
SUDS	−8.75	2.98	0.009	−15.04 to −2.45	
MyBodyPCA	13.65	6.46	0.050	0.02 to 27.28	
SUDS × MyBodyPCA	−5.42	1.99	0.014	−9.62 to −1.23	0.20

Overall goodness of fit F(3,17) = 4.36, P = 0.005, R-Squared = 0.41 (n = 21, one missing observation).

**Table 6:** Regression of *dSAMHappy* on SUDS and Visuomotor (Experiment 2).

Term	Coef.	Robust S.E.	P	95% C.I.	Cohen’s *f*^ 2^
Intercept	−1.16	0.85	0.187	−2.93 to 0.61	
Visuomotor (Async = 0,Sync = 1)	0.70	0.35	0.062	−0.04 to 1.43	0.23
SUDS	0.65	0.23	0.012	0.16 to 1.14	0.47

Overall goodness of fit F(2,19) = 6.09, P = 0.028, R-Squared = 0.38, (n = 22).

**Table 7:** Regression of *dSAMHappy* on SUDS and MyBodyPCA (Experiment 2).

Term	Coef.	Robust S.E.	P	95% C.I.	Cohen’s *f*^ 2^
Intercept	0.04	0.55	0.941	−1.11 to 1.20	
MyBodyPCA	0.43	0.12	0.003	0.17 to 0.69	0.84
SUDS	0.46	0.16	0.011	0.12 to 0.80	0.36

Overall goodness of fit F(2,18) = 12.06, P = 0.0005, R-Squared = 0.59 (n = 21, one missing).
