# Supplementary material for: Conversations between self and self as Sigmund Freud—A virtual body ownership paradigm for self counselling
Source: Sci Rep. 2015 Sep 10;5:13899. doi: 10.1038/srep13899 (PMC4564809; doi:10.1038/srep13899)
Supplement: Supplementary Information [file srep13899-s1.pdf]

Conversations between self and self as Sigmund Freud - A virtual body ownership paradigm for self counselling

Sofia Osimo, Rodrigo Pizzaro, Bernhard Spanlang & Mel Slater

Supplementary Information

1. Questionnaire Responses on Body Ownership and Agency

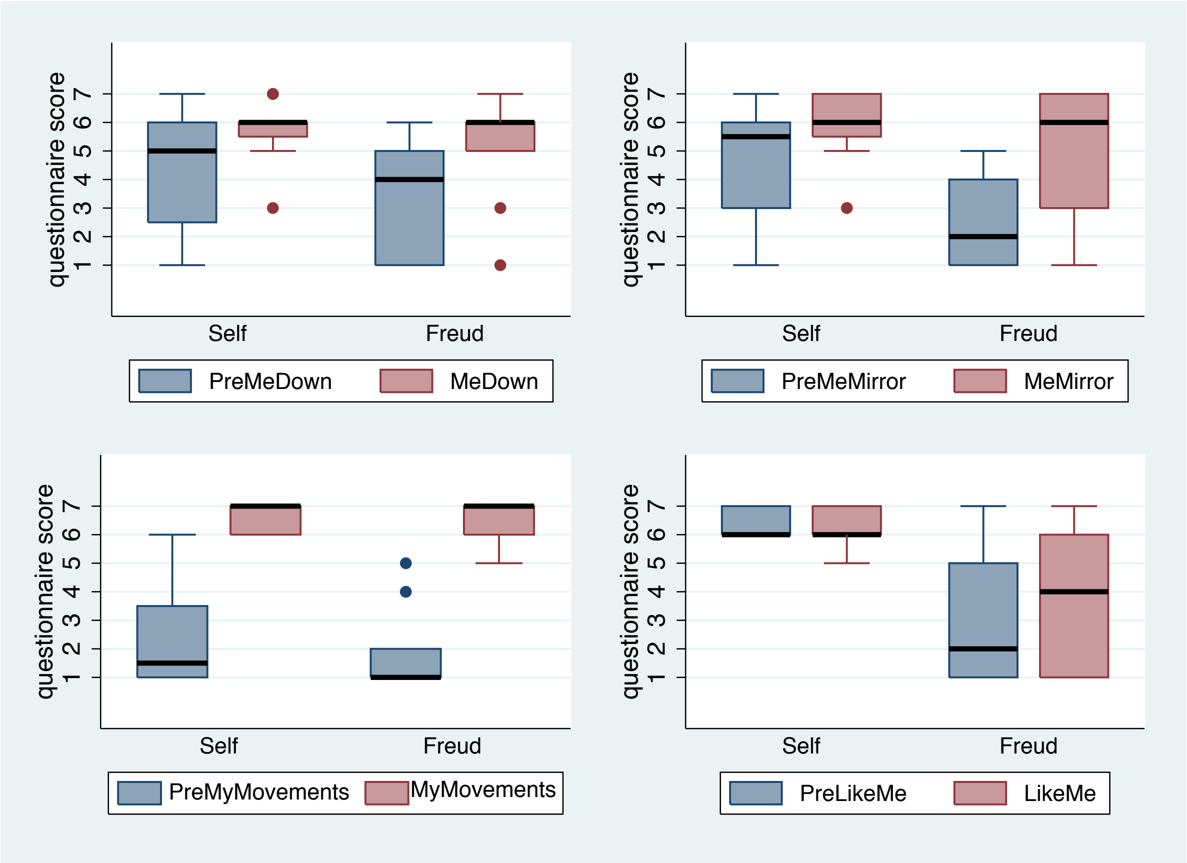

Supplementary Figure 1 - Box plots of body ownership questionnaire scores for experiment 1. The 'Pre' scores are asynchronous and the 'post' scores are synchronous. Using the Wilcoxon signed-rank test to compare the 'pre' (asynchronous) scores with the 'post' (synchronous) scores for each of Self and Freud we find: MeDown&Self: P = 0.016; MeDown&Freud: P = 0.005; MeMirror&Self: P = 0.010; MeMirror&Freud: P = 0.005; MyMovements&Self: P = 0.002; MyMovements&Freud: P = 0.003; LikeMe&Self: P = 0.564: LikeMe&Freud: P = 0.153.

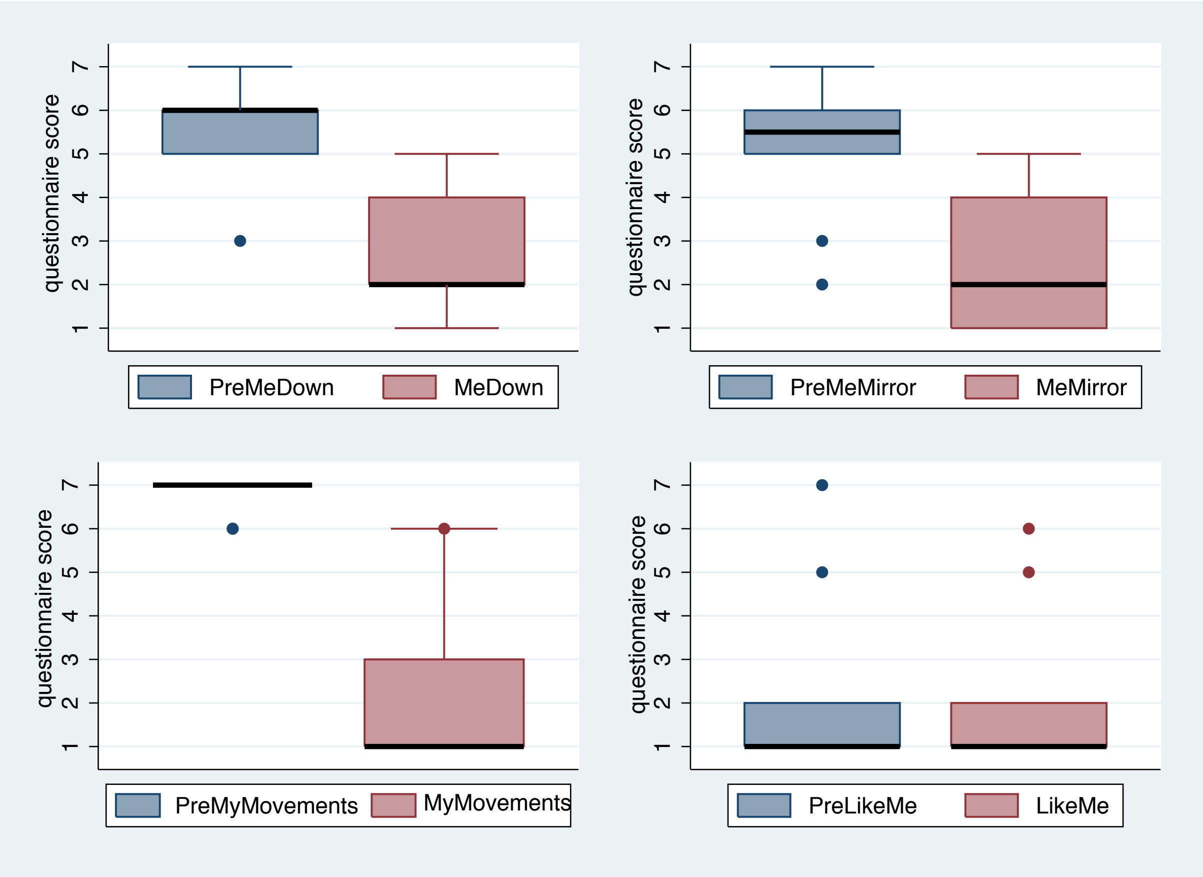

Supplementary Figure 2 - Box plots of the body ownership questionnaire scores for the asynchronous trials of experiment 2 (counsellor Freud). The 'Pre' scores are synchronous and the 'post' scores are asynchronous. Using the Wilcoxon signed-rank test to compare the 'pre' (synchronous) scores with the post (asynchronous) scores we find: MeDown: P = 0.006; MeMirror: P = 0.007; MyMovements: P = 0.004; LikeMe: P = 0.317.

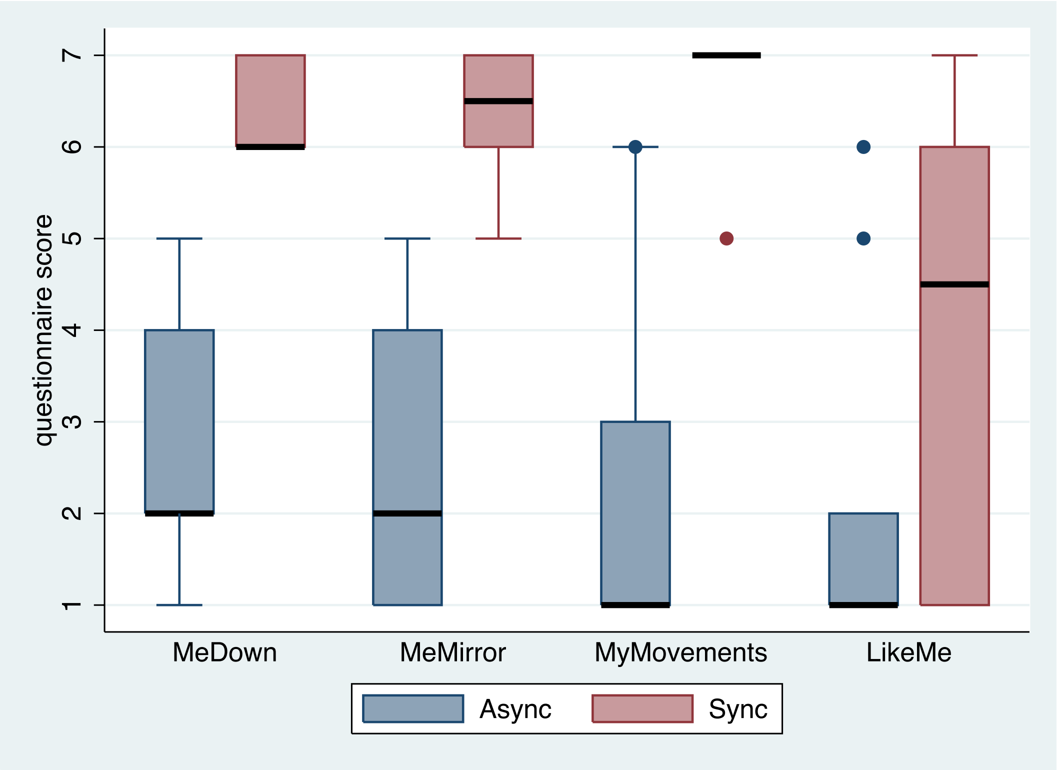

Supplementary Figure 3 - Box plots of the post questionnaire scores for the first trials of experiment 1 with the Freud counsellor, synchronous condition (n = 6) and the asynchronous trials of experiment 2 (Freud counsellor) (n = 10). Using the Wilcoxon rank-sum test to compare the scores between the two conditions we find: MeDown: P = 0.001; MeMirror: P = 0.001; MyMovements, P = 0.001; LikeMe: P = 0.120.

2. Evaluations of the Method

Various questionnaires apart from the POMS and SAM discussed in the paper were administered after the experience. This section presents each one and its results.

Supplementary Table 1 - Relationship to Problem

Median (Interquartile Range) of questionnaire responses. Participants responded to the following statements verbally immediately after the end of the experience, while still wearing the HMD. The responses were on a scale of 1 to 7 where 1 indicates complete disagreement and 7 complete agreement.

	Sync		Async
**Question statement**	**Self**	**Freud**	**Freud**
I feel that now I have more knowledge of my problem.	4(1)	5(3)	4(1)
I think that after the dialogue I'm capable to better understand my problem	5(2)	5(4)	4.5(2)
I believe that I can have new ideas about how to solve my problem	5.5(2)	5(5)	5(1)
I feel I can control my problem better	4.5(2.5)	5(3)	4.5(2)
Every time I changed my avatar and observed the situation from the perspective of the second avatar, I understood my problem better.	5.5(2.5)	5(3)	5(0)
This dialogue has helped me have a new perspective on my problem.	5(2)	5(3)	5.5(2)

Supplementary Table 2 - Session Rating Scale Evaluation - Means ± S.E.
Participants were asked to mark on a 10cm line their responses to each of the following questions.

I---I		Sync		Async
**Left Edge**	**Right Edge**	**Self**	**Freud**	**Freud**
I felt that I was not listened to, understood, nor respected.	I felt listened to, understood, and respected.	8.8 ± 0.28	8.5 ± 0.33	8.1 ± 0.39
We didn't work or talk about what I wanted.	We worked or talked about what I wanted.	8.9 ± 0.42	8.8 ± 0.30	8.3 ± 0.39
This therapy is not appropriate for me.	This therapy is appropriate for me.	8.2 ± 0.57	8.0 ± 0.75	6.9 ± 0.60
The session today failed.	In general the session today seemed good to me.	8.8 ± 0.43	8.1 ± 0.79	7.3 ± 1.02

Supplementary Table 3 - Recommendations Median (Interquartile Range)
Responses rated on a -3 to 3 scale with -3 representing Absolutely not, and 3 representing Absolutely yes.

	Sync		Async
**Question Statement**	**Self**	**Freud**	**Freud**
Would you recommend this experience to someone who had a similar problem?	2(2)	2.5(1.5)	3(1)
Would you do the same with another similar problem?	2(2)	2.5(2)	2(1)
Would you do the same with another more serious problem?	2(3)	1.5(2)	0.5(3)
Would you recommend to someone with a more serious problem?	2(1)	1.5(1.5)	1(4)

Supplementary Table 4 - Preference for Self or Freud as Counsellor (Experiment 1)
Frequencies in response to forced choice questions at the end of the second week

Question Statement	When I spoke with Self	When I spoke with Freud
Comparing this experience with the one last week which did you prefer the most?	4	8
Which for you was most useful?	6	6
Which would you recommend to a friend?	4	8

3. Participants

The 22 participants had mean (±SD) age 23.6 ± 6.1 (Asynchronous condition, n = 10) and 24.3 ± 4.8 (Synchronous condition, n = 12). 50% of them were students and the remainder various professional occupations (engineer, consultant, photographer, teacher) and 4 unemployed. The two groups were

similar in their level of computing and programming knowledge, previous experience of virtual reality, and computer game playing, as shown in Supplementary Table 5.

Supplementary Table 5 - Medians (IQR) of questionnaire responses on computing knowledge, virtual reality experience and videogame playing

**Question**	**Async (n=10)**	**Sync (n=12)**
Please indicate your level of knowledge of computing on a scale of 1(none) to 7 (expert).	4(2)	4(1)
Please indicate your level of knowledge of computer programming on a scale of 1(none) to 7(expert).	1.5(2)	1(0.5)
Have you had previous experience of virtual reality on a scale of 1(never) to 7(extensive experience)	1(5)	1(2)
With what frequency do you play video games 1 (never) to 7 (always)	5(2)	4(2.5)

4. Supplementary Video S1

This video shows an illustration of the complete experiment.

The music is:
"Dream Culture" Kevin MacLeod (incompetech.com) Licensed under Creative Commons: By Attribution 3.0 http://creativecommons.org/licenses/by/3.0/
